# British Medicinal Plants

**Published:** 1892-01

**Authors:** Alfred Heath

**Affiliations:** London, England


### BRITISH MEDICINAL PLANTS.

ALFRED HEATH, M. D., F. L. S., LONDON, ENGLAND.

### ORDER 37.—UMBELLIFERÆ. (CONTINUED.)

*Cicuta virosa* (Cowbane, Water Hemlock).—This plant does not appear to have been much used in medicine; probably on account of the danger in taking so active a poison. There is no question that it is a marvelous medicine in diseases of a spasmodic character, and thanks to the provings of the plant and its consequent introduction into the homœopathic materia medica, many extraordinary cures of epilepsy have been made. I remember a case of a woman I attended some years ago, who was in apparently good health, when she suddenly fell down in a violent fit of epilepsy, with tonic and clonic spasms of the whole body, at one time fighting in the most furious manner, and then every fibre set fast like steel; perfect insensibility for six hours in spite of everything that was done by myself and others. While sitting by her side she suddenly began to shake and jerk one arm. This made me think of Cicuta. I immediately dropped a few drops of a low tincture between her teeth. About four decimal strength I believe it was. The result was magical. In ten minutes she completly recovered sensibility. She had no other medicine after this, and has never had another attack to this date.

In England this plant is both local and scarce, and it often confounded with *Œnanthe crocata* and *Œnanthe phellandrium* by ignorant collectors. It is said to lose much of its medicinal activity when dried, therefore the tincture should be made from the freshly gathered roots or plant.

This drug produces violent spasms of almost every part of the body. Spasms and swelling of the tongue, spasms of throat on swallowing, startings, jerkings, twitchings, tremblings, etc., spasms of the jaw, involuntary urination, convulsions, violent and exhausting spasms of the muscles of the neck; spasms of the back, drawing the back backward like an arch, complete loss of power, contortion of the limbs, jerking, staggering, tetanic stiffness of back and legs, and a host of other similar symptoms.

*Apium graveolens* (Wild Celery).—Common in marshes and ditches, especially near the sea. The plant is acrid and poisonous, but when cultivated it loses its poisonous properties, and is eaten as a salad. It has been used as a diuretic, and for gravel, also in jaundice and obstruction of the liver and spleen, and for colic. There is some proving in Allen's *Materia Medica.*

*Apium petroselinum, Petroselinum sativum* (Common Parsley).—This plant is a native of Sardinia, but is quite naturalized in our gardens. Apiol lately come into use as an emmenagogue is obtained from it. The juice of this plant has been used with some success in allopathy against ague; also as a remedy for ring-worm. The root is aperient and diuretic, and has been employed in nephritic pains and obstruction of urine. Allen's *Materia Medica* has some proving.

*Ægopodium podagraria* (Gout Weed).—The root and leaves of this very common English plant have been used externally in all kinds of pains, sciatic and other nerve pains. The plant has obtained its name from its singular efficacy against the pains of gout, but I need not say that external applications for relieving the pain of gout are also singularly dangerous, and I know of valuable lives that have been lost within an hour by such unscientific practice. Probably by proving the plant we should find that it is homœopathic to gouty pains, and if so an infinitesimal quantity taken internally would safely and effectually remove the tronble.

*Carum carvi* (The Caraway Seed).—This plant is cultivated in boxes, and is found wild in other parts. Although not strictly British the caraway seed is mentioned in works of English botany. The seeds are used as condiments and contain a volatile oil. The plant obtains its name from Caria, a province in Asia, where it grows abundantly. The seeds are carminative, cordial, and stomachic and are used in dyspepsia, flatulencies, hysterical and hypochondrical disorders. There is no proving.

*Pimpinella Saxifraga* (Barnet Saxifraga).—This plant at one time had a place in the Edinbugh Pharmacopœia, and was used as a diuretic, and against gravel. German physicians have used it in removing tumors and obstruction of glands and in cutaneous diseases; also in paralysis of tongue. There is no proving.

*Œnanthe crocata* (Water Dropwort).—This plant does not appear to have been much used in medicine until it was introduced into homœopathic practice. Many lives have been lost by ignorant people mistaking it for parsnips, etc. Cattle also are frequently poisoned by eating it. The proving of the drug shows that it is a medicine that produces all kinds of convulsive spasms like epilepsy, tetanus, spasms, with loss of consciousness, foaming at the mouth. Many wonderful cures of epilepsy and other convulsions have been made with this valuable drug. There is a short proving in Hering's *Guiding Symptoms.*

*Œnanthe phellandrium* (Fine-leaved Water Hemlock).—Known in the homœopathic materia medica as *Phellandrium*

*aquaticum.* This is not a very common plant in this country, and moreover it is very local. Horses are said to have become paralyzed after eating it, owing it is believed to a small beetle that lives on it belonging to the *Curculionidœ* and called *Lixus paraplecticus.* The seeds of Phellandrium were said by Pliny to be efficacious in calculous complaints, and other disorders of the bladder, it also is diuretic and emmenagogue, it has been successfully employed in the treatment of wounds and inveterate ulcers, and even in cancer, in phthisis, asthma, dyspepsia, and intermittent fevers. There is some proving of the symptoms in Hering's *Guiding Symptoms.* It causes urging to urinate with *scanty* discharge, hence its power to act as a diuretic. It produces, and is therefore homœopathic to pains in the nipple of of an intolerable severity, great pains in the breast, sore nipple, with purulent discharge, loss of strength, with mental distress and spells of hysterical weeping, with painful stitches under the breast; also continuous cough for an hour or more, accompanied with shortness of breath and prostration, cough dry, with suffocation, frequent easy expectoration, spasmodic cough, with emaciation, bronchial cough, loss of appetite, sleeplessness, swelling of the hands, profuse sweat, diarrhœa, vomiting, copious, purulent sputa, emaciation. This drug has been used homœopathically with success in phthisis; also in last stages, when expectoration has become terribly offensive. It produces chills every third day at four P. M., continuing one hour, and preceded by severe rheumatic pains in the arms, fever and sweat lasting two hours, followed by violent headache, nausea, and stitching pains in the feet; generally the day after these symptoms entire inability to urinate.

